# Supplementary material for: Glass factory found: Basinwide (600 km) preservation of sponges on the Phosphoria glass ramp, Permian, USA
Source: PLoS One. 2025 Nov 12;20(11):e0333211. doi: 10.1371/journal.pone.0333211 (PMC12611142; doi:10.1371/journal.pone.0333211)
Supplement: S3 File — (DOCX) [file pone.0333211.s003.docx]

**REFERENCES FOR SUPPORTING INFORMATION**

48. McKelvey, VE, Davidson, DF, O’Malley, FW, and Smith, LE. Stratigraphic sections of the Phosphoria Formation in Idaho, 1947-48, Part 1. U.S. Geological Survey Circular. 1953. Report No.: 208.

49. Smith, LE, Hosford, GF, Sears, RS, Sprouse, DP and Stewart, MP. Stratigraphic sections of the Phosphoria Formation in Utah, 1947-48. U.S. Geological Survey Circular. 1952. Report No.: 211.

50. O’Malley, FW, Davidson, DF, Hoppin, RA, and Sheldon, RP. Stratigraphic sections of the Phosphoria Formation in Idaho, 1947-48, Part 3. U.S. Geological Survey Circular. 1953. Report No.: 262.

51. McKelvey VE, Armstrong, FC, Gulbrandsen, RA, and Campbell, RM. Stratigraphic sections of the Phosphoria Formation in Idaho, 1947-48, Part 2. U.S. Geological Survey Circular. 1953. Report No.: 301.

52. Sheldon, RP, Warner, ME, Thompson, ME, and Peirce, HW. Stratigraphic sections of the Phosphoria Formation in Idaho, 1949, Part 1. U.S. Geological Survey Circular. 1953. Report No.: 304.

53. Davidson, DF, Smart, RA, Peirce, HW, and Weiser, JD. Stratigraphic sections of the Phosphoria Formation in Idaho, 1949, Part 2. Geological Survey Circular. 1953. Report No.: 305.

54. Cheney, TM, Smart, RA, Waring, RG, and Warner, MA. Stratigraphic sections of the Phosphoria Formation in Utah, 1949-51. U.S. Geological Survey Circular. 1953. Report No.: 306.

55. Smart, RA, Waring, RG, Cheney, TM, and Sheldon, RP. Stratigraphic sections of the Phosphoria Formation in Idaho, 1950-51. Geological Survey Circular. 1954. Report No.: 327.

56. Rios, N. E. & Bart, H. L. GEOLocate (Version 3.22) [Computer software; accessed January 2024]. Belle Chasse, LA. 2010. Tulane University Museum of Natural History.
