# Supplementary material for: Glass factory found: Basinwide (600 km) preservation of sponges on the Phosphoria glass ramp, Permian, USA
Source: PLoS One. 2025 Nov 12;20(11):e0333211. doi: 10.1371/journal.pone.0333211 (PMC12611142; doi:10.1371/journal.pone.0333211)
Supplement: Attachment — Submitted filename: PONE-D-25-21229 Reviewer Comments.pdf [file pone.0333211.s004.pdf]

Review of PLOS One – PONE-D_25-21229 — Glass factory found: Basinwide (600 km) preservation of sponges on the Phosphoria glass ramp, Permian, U.S.A.

The manuscript by Wistort et al. submitted to PLOS One combined novel original lab/field work and interpretation with a detailed literature review. The authors propose a novel interpretation for a series of large-scale tubular features found throughout chert-bearing members of the Phosphoria Rock Complex. The authors interpretations are scientifically robust and sound and they are a new useful interpretation of these features in the emerging concept of glass ramps. As this is the core tenet of the manuscript, I commend the authors for this. As a result of this, I believe this paper is a valuable contribution to the literature on both the Phosphoria and glass ramps and look forward to seeing it published.

That being said, while the manuscript is generally well written, sufficiently organized and includes appropriate figures, there are a number of sections I believe the authors could 'clean up'. Most of these involve relatively minor changes including adding additional detail, more appropriate use of citations, addressing of a few aspects of their model that aren't addressed, fixing mistakes on figures, and clarifying sections that are not overly clear or have ambiguity. There are also a few aspects of the paper where I believe the authors need to revisit their data/figures in a more significant way, primarily regarding the lack of data in part of the landward Phosphoria Sea, as well as in the stratigraphy/age model used. Neither of these take away from or will modify the conclusions of the manuscript but will strengthen the robustness of the supporting data and background.

I believe that these suggestions will result in a 'moderate' amount of work to address these issues and look forward to seeing this work published in a stronger and more robust paper. I encourage the authors to view comments my comments as constructive criticism that is meant to help improve the manuscript that accompanies strong underlying scientific motivations and conclusions. I have provided extensive comments below.

Best,
Edward Matheson

General Comments

1) I note that some of the authors' citations do not really match the text they accompany. Appropriate citations for these instances are cited elsewhere and so this is clearly a relatively minor oversight. This isn't a major issue but I would encourage the authors to clean up their citation usage as it currently stands. For example:
    - Lines 33-35 – Refs 2-7 are largely 1950s to 1980s papers with 1 exception (2004) and largely relate to the first half of the sentence "vital economic resource…" None relate to the position/existence of the PRC at the southern end of widespread

epicontinental chert deposition. There are numerous citations to this effect, they are just in later sentences.

- Line 41 – Citation 15 is the Geologic History of Utah. This does not seem an appropriate reference for the sentence before. There are numerous theses, conference presentations, peer-reviewed papers, etc. that deal explicitly with the cherty members/formations of the PRC. Many are cited elsewhere so this seems like a minor (e.g., Ritterbush, 2019; Matheson and Frank, 2020; Wistort et al., 2022).

Moderate/Major Comments

2) Line 41-43 – "Previous researchers…" – This is a truthful statement but for the reader not familiar with glass ramps, it may be confusing. First off, you have only mentioned phosphorites and chert in the PRC up until this point. You have not mentioned any carbonates. It might be worth indicating that there are also thick carbonate packages in the PRC at times when there is not phosphogenesis. Secondly, this statement/section would benefit from the explicit discussion of the other side of the equation, which is not just why there was minimal carbonate production but also why there wasn't phosphogenesis, and likewise, why siliceous sponges dominated. A lack of carbonate producing benthos throughout much of the rock record produces hardgrounds, omission surfaces, transgressive lags, or various other deposits/surfaces that resulted from sedimentary condensation in carbonate environments (cf. https://doi.org/10.1016/j.earscirev.2015.11.016). So some more details here are needed either to clarify that you are not just talking about 'low carbonate production' (line 42) but the dominance of a biosiliceous biota over a biocalcifying benthic biota, potentially with some detail. You may also refer to later in the manuscript for greater detail, but as it stands, this section feels incomplete/misleading due to omission.

3) Figure 1D – Why is there no data for the Bighorn Basin of north-central Wyoming? The PRC exists here and the Tosi is widespread across the area. That the grey polygon does not extend into this area and that there is no data from up here is worrisome as it casts doubt on the comprehensiveness of the thoroughness of the data used. See Matheson and Frank (2020b; Sedimentology), which is cited in this paper, Figures 9 and 10b (as well as many others) which are maps and fence diagrams of the Tosi's existence and hundreds of wireline logs, cores, and outcrops used to constrain it. See also Matheson and Frank (2020a; Sedimentary Geology) which focuses purely on the Tosi Chert in the Bighorn Basin. This relevant paper entitled "An epeiric glass ramp: Permian low-latitude neritic siliceous sponge colonization and its novel preservation (Phosphoria Rock Complex)" is not cited in this paper about chert in the Phosphoria Rock Complex. Other references (e.g., Coalson and Inden, 1990) include mention of the Tosi Chert in the same area as well and might be relevant. Surely the data from these, even if not

paleontological is useful to constrain the hydrodynamic zonations, presence/absence of the tubular fossils, etc.

4) Lines 57-62 – You begin by stating that the volume of sponge spicule-rich rock retained in the PRC presents a puzzle for two reasons. The information in this paragraph is valid, but not logically connected and could be clarified. It takes a few reads to fully understand the points. I think you need to reword this statement as it is confusing. It sounds like you are stating that the puzzling aspect is that there is such a large volume of "sponge spicule-rich rock". Is it simply the volume that is the puzzle? Or is it something more? Is it that the volume indicates the predominance of sponge-dominated communities for long periods? Is it that the spicules are preserved and not diagenetic altered/destroyed? Is it that the spicules are preserved as disarticulated spicules with relatively few in situ sponges? There are elements of this paragraph that would indicate it is each of these three things that is puzzling, but they are very intermixed. Perhaps, you might reword to something like "The sheer volume of sponge spicule-rich rock in the Phosphoria is atypical and leads to a number of unanswered questions that remain puzzling. First…" and then explicitly list what is puzzling and why.
   a. Reason 1 that is provided is that spicules disarticulate, but this does mean they aren't present/retained. You then mention diagenesis but don't clearly articulate why this should mean that there aren't large volumes of spicule-rich rocks preserved. Diagenesis can act to further 'preserve' depositional textures even if the primary fossils are not preserved, as is also common in carbonates (e.g., molds, neomorphism, ghosts). So, arguing that spicules undergo diagenesis isn't necessary an argument against spicule rich rock. Even if the diagenesis is fabric destructive, you clearly state that the silica remains as chert. So, the reader might interpret your argument as something to the effect of "we often know there were likely lots of spicules, but they aren't visible in the chert, and so the fact that they are preserved here is a puzzle". Is this what you are saying? If not, consider rewording to clarify this argument and its relation to the main point made in the first sentence of the paragraph.
   b. Your second reason is that "degraded and detrital spiculites reveal few clues about the sponges' original habitat". This is not a puzzle presented by "the sheer volume of sponge spicule-rich rock" but by the fact that there are relatively few in situ sponges, which is not what the first sentence would indicate. Again, I think the ideas in this paragraph are good, but somewhat disorganized and could be clarified so the reader fully comprehends the outstanding questions.

5) Lines 75-76 – There are some major questions/clarifications needed regarding your stratigraphy and age model. While a minor part of the paper, these should be cleaned up. For example, you indicate uncertainty in the age of the PRC, indicating 'possibly late Permian' as if it is less likely.

c. First off, you cite Wardlaw and Collinson (1986) regarding the age, which is a good reference, but is relatively old. It is not completely wrong but I'd encourage you to cite more recent work include Bruce Wardlaw's 2015 paper before he passed (https://www.jstor.org/stable/44511382). There are other references that would also supplement, some peer-reviewed, others in the grey literature (theses, USGS reports) as well, cited in the above Wardlaw (2015) paper as well as the numerous thesis I refer to in the subsequent points below.
d. Furthermore, on Figure 2 you have modified the original Cressman and Swanson (1964) figure and added ages and adopted the age model where the PRC stretches into the late Permian, presumably based on Davydov's data, which you cite in text. The Late Permian age was not the prevailing model when Cressman and Swanson published their correlation chart. At the very least, you have not indicated that on the caption for Figure 2 that this is the age model you are presenting and the source.
e. Related, you have not fully adhered to the Davydov model. Davydov (2018) Figure 6 for example puts the Rex Chert and Gerster at Lopingian. You have the Rex Chert as various ages, but mostly within the Guadalupian. Davydov's age dates came from the Meade Peak below the Rex Chert and put it at latest Guadalupian/Capitanian. This is not what you show.
f. Secondly, you have retained the Cressman and Swanson stratigraphic relationships, which are 60+ years old. They aren't entirely what Davydov or others would/do propose, so you'll need to justify combining two different stratigraphic and age models.
g. And the figure (Cressman and Swanson's stratigraphy) shows that the 3 sequence/cycles (Grandeur, Franson, and Ervay) are partially overlapping temporally (e.g., the Ervay cycle in MT started when the Franson cycle was still going on in WY, etc.). We now think of these three cycles as being unconformity (and correlative conformity) bound packages of genetically related strata (i.e., sequences) caused by relative sea level changes. As such, having overlap (of millions of years) of the end of one cycle in one part of the sea with the beginning of the next cycle in other parts of the sea is seemingly unlikely. The Cressman and Swanson (1964) stratigraphic model was published prior to sequence stratigraphy and our understanding of the basin and Phosphoria stratigraphy has evolved. This is not to go into the degree of diachroneity implied by the varying ages for the same stratigraphic members in various portions of the Phosphoria Sea implied by the Cressman and Swanson figure, which is again not fully consistent with the current consensus.
h. I would encourage the authors to rethink their stratigraphy in Figure 2. While I recognize that this is not a major part of the paper, adopting a 60-year-old stratigraphy and fitting it into a recent age model (and one that is highly contentious at that) based on a different stratigraphy takes away from the true

content of the manuscript. I would refer the authors to the various literature that has been published since the original sweet of USGS reports that they cite. This includes recent work (last 10 years) and intermediate age work (80s-2000s). For example:

i. Max Pommer (e.g., https://doi.org/10.2110/sepmsp.112.13 and https://www.proquest.com/openview/7dc568b8b93f5d2cc0a0ff7df554a6a8/1?cbl=18750&pq-origsite=gscholar)
ii. Edward Matheson (already cited)
iii. Madeline Marshall (already cited)
iv. Piper et al. (2007) - dx.doi.org/10.1016/j.dsr2.2007.04.012
v. Hiatt and Budd (2003) - already cited
vi. Hendrix and Byers (2000) - https://doi.org/10.2110/pec.00.66.0349
vii. Whalen (1996) - https://archives.datapages.com/data/rocky_sepm/data/034/034001/355_rocky_mount340355.htm
viii. Maughan (1984) - GEOLOGICAL SETTING AND SOME GEOCHEMISTRY OF PETROLEUM SOURCE ROCKS IN THE PERMIAN PHOSPHORIA FORMATION
ix. Peterson (1984) – PERMIAN STRATIGRAPHY, SEDIMENTARY FACIES, AND PETROLEUM GEOLOGY, WYOMING AND ADJACENT AREAS.

i. The PRC stratigraphy is a mess but I would advise you to do some thinking about what age and stratigraphic model to adhere to and perhaps provide some clarification as to that in the text or figure caption.

6) Lines 94-101. This paragraph seems like it would be a better fit for the closing paragraph of the Introduction, as it explains the main focus of this study. It could fit well prior to the sentence "Herein, we argue…." That starts on Line 66.

7) Methods –
   a. It is mentioned that data is collected from regional surveys (previous studies), which is great and S1 and S2 support this well. However, it is also mentioned that there are "recent field and laboratory observations" and Figure 2 indicates that the authors visited sites in UT and WY. However, I do not see those sites listed in S1 as being visited by the authors. Wistort et al. (2022) is listed as visiting sites in UT and NV, but not WY. I would like to see the authors explicitly lay out where their samples are from, what sites, whether they were visited as part of this project or are using samples from earlier work (e.g., "samples collected during the study in XXX et al. (YYYY)"), numerous of stratigraphic units covered by their samples, etc.
   b. It is mentioned that thin sections were cut, but how many? Again, more detail on the actual dataset would be used. And what type of equipment was used and methods? Just standard transmitted light or was any white card petrography

done, what about any cathodoluminescence or fluorescence, or SEM work? Details on how the thin sections were analyzed would be useful.

   c. Line 122 - “Facies analysis was determined”. What facies analysis? Was this a lithofacies analysis or a microfacies analysis or some other kind? I see description of chert concentrations in the results but no evidence of a facies analysis with description and interpretation of facies? What is meant by this?

8) Results – Some questions arise when reading this section:
   a. Line 135 – Any descriptions of the bases or the bedding plane/horizon they are found on? Are bases all at the same level, or randomly throughout the stratigraphy, etc.?
   b. Line 136 – Can you verify that all of these features are 5-6 cm in diameter? None larger, none smaller?
   c. Line 142 – If beds are 6 meters thick, and the tallest extends vertically for 1.5 m (line 136), are there multiple above each other? What are the relationships between different levels/horizons/individuals?
   d. Line 149 – Define ‘mutual contact’ for those readers not familiar. Also, clarify what is meant by “they” as this is the beginning of a new paragraph.
   e. Line 150-151 – How is the matrix described here different from that described on Lines 142-143? Why is this information separated?
   f. Line 156 – “Zebra-Textured Diagenesis” – It is not the ‘diagenesis’ that is zebra-textured (diagenesis is a process), but instead the chert, correct?
   g. Line 166 – What evidence is there for “void-filling chalcedony”? Determine whether chalcedony/chert is cement vs. replacement can be challenging so evidence (and a figure?) demonstrating this would be beneficial.

9) Discussion – I am in general agreement with much of the Discussion. This is a well-versed portion of the manuscript. A few comments/thoughts:
   - There is no mention of inclusions in the silica in any component of the tubes/sponges. Could this not be useful information validate or disprove your model?
   - Why are they all the same size (5-6 cm)? This is not further discussed? Is there anything from modern sponge communities or your observations which might indicate a reason and whether this is meaningful?
   - What is the zebra texture present in the outer core? Why is it present? There is no mention of the paragenesis here and what it might indicate?
   - You have relatively minimal description of the chert fabrics. Additional description might strengthen the interpretations.
   - Where do the phosphatic peloids in some of the cores come from? This is described but not revisited in detail.

Minor Comments – Text and Figures

10) Line 40 – "the Phosphoria" – You use this term to refer to the Phosphoria Rock Complex (as defined on line 32). On line 40, you mean the Phosphoria Sea, not the Phosphoria Rock Complex. The group of rocks that was produced from the sediments did not favor the prolific growth of sponges, but the depositional basin that the sediments were accumulating it (and it's chemical/physical oceanographic parameters) did.

11) Lines 41-42 – "Previous researchers postulate" - Tense is off here. "Either previous researchers postulated" or "Some researchers postulate" or something to that effect

12) Lines 73-75 – You refer to the Formation names in the text and refer to Figure 2. Very few of the formation names are actually indicated on Figure 2. You simply have lithologies and sometimes the formation/group names for each member.

13) Line 88 – Would https://doi.org/10.1016/j.sedgeo.2019.105568 not be better than reference [24], given that it focuses exclusively on the glass ramp and spicule-rich facies

14) Line 112 – Figure 3 caption - "Thin section" - Should be plural. Should also be "photomicrographs" as they are images of thin sections, not the thin section itself.

15) Line 213 - "sponge colonize" should read "sponges colonize the…"

16) Line 214 – "outer most" vs. "outermost"

17) Line 259 - No citation for the source of these observations?

18) Line 276 – "more specifically," – this would imply that the Phosphoria Sea phosphorite is a component of the Permian Chert Event. I think this requires rewording to clarify what you mean.

19) Line 277 – Colon incorrectly used as it does not follow a complete sentence.

20) Figure 1 –
- Citation for the chert belt and paleogeography in panel A and panel B is missing.
- Panel B refers to "white numbers" – none are present on the figure
- Nevada is labelled incorrectly in Panel B.
- The 10 N latitude line in Panel B does not match with the latitude shown on Panel A, which is 20-30N.

- Citations for the sources of data for the grey polygons in panels C and D would be useful.
- Caption – “Rex Chert” and “Tosi Chert” are proper lithostratigraphic names if the word “chert” is capitalized. As such the sentences should be “approximate extent of the Rex Chert” (note ‘the’ added). If you are referring to chert of the Rex, then no ‘the’ is needed but chert should not be capitalized.

21) Figure 2
- See comments above regarding validity of the Cressman and Swanson stratigraphy from which this figure was derived, proper attribution of the source of the age model, etc.
- On lines 73-75 – You refer to the Formation names in the text and refer to Figure 2. Very few of the formation names are actually indicated on Figure 2. You simply have lithologies and sometimes the formation/group names for each member.

22) Figure 3
- Panels H and I are not described per se. There are brief indications of the cut and location.

23) Figure 4
- What does the text “Variations in morphology in Utah and Wyoming specimens” refer to? I see no representation of such variation in the figure.

24) Figure 5
- Caption does not indicate whether this is Rex or Tosi time, just “Phosphoria Basin”? Looking back, the data included seems to indicate Rex time?
- You refer to the ‘Phosphoria Basin’ as ‘Phosphoria Sea’ elsewhere.
- Inconsistent capitalization of the word ‘zone’
- The high energy zone is described as white in the caption but it is actually light blue
- What is the dark blue that is in Montana and Utah and Nevada and equivalent to the light blue high-energy zone? This is not discussed at all.
- Re-add the caption or a legend describing the difference between blue and black dots so readers don’t have to scroll all the way up. That data is important as it informs how you identified your zones.

25) S1 – Andersson and Sauvagnat (1988) is improperly listed as Andersson Sauvagnat (without the ‘and’) multiple times.
